# Global prevalence and incidence of chronic subdural hematoma: A systematic review

**DOI:** 10.1016/j.bas.2025.105893

**Published:** 2025-12-01

**Authors:** Azra Dziho, Abdullah Al Awadhi, Caterina Mollica, Emily Richards, Rohan Sanghera, Alex Fleet, Angela Huttner, Karl Schaller, Simone Grannò, Aria Nouri

**Affiliations:** a Division of Internal Medicine, Department of Medicine, Neuchatel Hospital Group, La Chaux-de-Fonds, Switzerland; b Division of Neurosurgery, Department of Clinical Neurosciences, Geneva University Hospitals, Geneva, Switzerland; c Faculty of Medicine, University of Cambridge, Cambridge, United Kingdom; d Clinical Research Center, Geneva University Hospitals, Geneva, Switzerland

**Keywords:** Chronic subdural hematoma (cSDH), Global incidence, Aging population, Antithrombotic therapy, Traumatic brain injury

## Abstract

**Introduction:**

Chronic subdural hematoma (cSDH) is one of the most common conditions in neurosurgery. However, its epidemiology remains poorly investigated.

**Research question:**

To systematically evaluate all available evidence on the global prevalence and incidence of cSDH from 1970 to 2022.

**Methods:**

A systematic review according to PRISMA guidelines using PubMed, Cochrane, and EMBASE was conducted on articles from 1970 to 2022. Studies reporting regional-to-national incidence or prevalence were included. Studies were graded based on quality of evidence.

**Results:**

6253 articles were identified, 18 meeting inclusion criteria. Seven articles reported surgical incidence and 11 provided incidence based on imaging or clinical findings. Six additional articles were evaluated for demographic data. No study discussing prevalence was found. 18 reported incidence from 12 countries in five continents. The lowest incidence was found in Brazil (3.39/100,000/year), and the highest in the USA at 39.1/100,000. Incidence increases with age and may be up to three times higher among patients over 80. It also appears to increase over time, probably with improved diagnostics. In most studies, incidence was higher in men. The most common aetiology was trauma and falls. Other contributing factors were chronic alcohol abuse, anticoagulation, and violence. Several articles did not report any cause.

**Discussions and conclusions:**

Epidemiological data remain sparse, with limited incidence data and no prevalence data. The level of evidence remains medium to low with regional differences in methodology, suggesting the need for standardisation. Incidence has increased over time in all regions, particularly amongst older patients. This trend will likely continue with an ageing population.

## Highlights

• First systematic review on global epidemiology of chronic subdural hematoma.
• No prevalence data; incidence reported in 12 countries across 5 continents.
• Incidence 3.4–39.1/100,000 yearly, higher in men and elderly patients.
• Rising incidence linked to aging, trauma, and antithrombotic medication use.
• Methodological heterogeneity shows need for standardized epidemiology.

## 1 Introduction

Chronic subdural hematoma (cSDH) is one of the most frequently encountered conditions in neurosurgery worldwide (Welling et al., 2018). Although a standard CT scan is sufficient for diagnosing cSDH, the incidence is influenced by various factors, including patient demographics, access to healthcare and awareness of symptoms.

cSDH consists of a blood collection in the cranial subdural space, believed to originate from bridging veins, draining blood from cortical veins towards the cerebral venous sinuses, typically after a relatively mild cranial trauma. While the pathogenesis remains incompletely understood, it is thought that the blood clot formed from the initial hematoma liquefies, and stimulates a local inflammatory reaction resulting in pseudo-membrane formation and slow mass expansion (Nouri et al., 2021; Sahyouni et al., 2017). The inflammatory process is believed to be accompanied by disorganized fibrinolysis and angiogenesis resulting in the formation of leaky blood vessels (Nouri et al., 2021; Edlmann et al., 2017a). Regardless of the underlying mechanism, the progression of cSDH ultimately leads to cerebral compression and irritation which, when symptomatic, necessitates surgical evacuation. Known risk factors include coagulopathies, intracranial hypotension (Yadav et al., 2016), chronic alcoholism (Chen and Levy, 2000), the use of anticoagulant and antiplatelet medication (Sim et al., 2012) as well as cortical atrophy (Yang et al., 2012).

As cSDH is prevalent in the neurosurgical field, its economic impact is significant, but unfortunately underappreciated. It has been estimated that in the USA, the mean operative cost per patient ranges from $7588 for burr-hole evacuations to $10,416 for craniotomies (Regan et al., 2015). When considering the overall episode of care, the total cost averages $26,659 for surgically treated patients and $10,234 for medically managed patients (Hendrix et al., 2022). Despite its high frequency, and the likelihood of rising incidence due to population ageing and increased use of antithrombotic medications, there has been no coordinated effort to describe the global epidemiology of cSDH. The aim of this systematic review is therefore to provide a comprehensive overview of the worldwide epidemiological landscape of cSDH. Specifically, we summarize the available literature on the global incidence and prevalence of the disease, as well as its main epidemiological determinants, including age, sex, and underlying causes.

From a global perspective, the review will help identify regions with and without data due to under-diagnosis, assess regional variations, determine the global burden of the disease, and estimate increases in costs associated with demographic changes.

## 2 Methods

### 2.1 Search strategy and selection criteria

A systematic review in accordance with the PRISMA-P (Preferred Reporting Items for Systematic Reviews and Meta-Analyses) guidelines as depicted in the PRISMA Statement undertaken from February to August 2022.

The literature research was conducted using PubMed, Cochrane, and EMBASE to collect articles published between 1970 and January 2022, from the time the CT-scan was globally introduced to the time we conducted the research. The included keywords were the following: “Chronic Subdural Hematoma”, “Incidence”, “Prevalence”, “Epidemiology”, “Demographics”, and “Surgery”. All languages were included for review. Articles that did not distinguish the types of subdural hematomas (i.e., acute, subacute, or chronic), case reports, case series, meeting abstracts, literature reviews, editorials, animal studies, papers that discussed cSDH in conjunction with a specific comorbidity or treatment or other non-general population cohorts, and cSDH in patients under the age of 18 were excluded.

All articles meeting the initial search criteria were reviewed by two authors. A third reviewer resolved any potential conflicts where there was no consensus. Where provided, the regional, provincial/state, or national prevalence or incidence of cSDH were searched and extracted. From the incidence studies, the definition of cSDH, method of data collecting, reported incidence, male-to-female ratio, age of highest incidence, mean age, and the cause of cSDH were extracted. For prevalence studies, the initial objective was to extract the definition of cSDH, the prevalence calculation, and the reported prevalence.

**Fig. 1:**
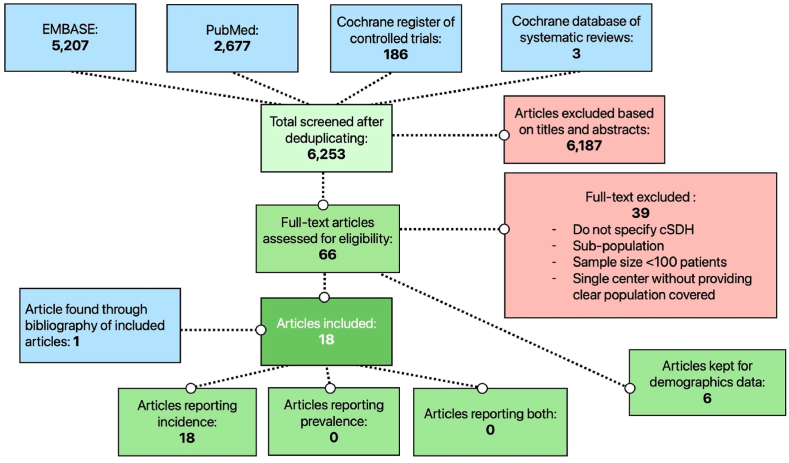
Study selection (PRISMA Flow Chart).

The PRISMA flowchart is reported in Fig. 1 and the literature review has been evaluated using AMSTAR 2 tool, reported in Supplementary material 3.

### 2.2 Data analysis

We extracted seven variables from each included article and 4 in articles analysing incidence in subgroups, listed in Table 3a, Table 4aa and Supplementary material 1. Two of the authors further graded the articles based on their level of evidence using an adjusted scale developed by *Singh* et al. in an article discussing the epidemiology of traumatic spinal cord injury (SCI) (Singh et al., 2014).

We adapted this scale for our review by removing the item on reliability, as none of the included studies reported this measure. We also converted the original qualitative interpretation into a quantitative scoring system by assigning points to each article. The specific questions and the corresponding scoring criteria are presented in Table 1. When the two reviewers disagreed on the level of evidence, both ratings were reported.(see Table 3b)

**Table 1:** Scale and interpretation Scale used to grade included articles based on their level of evidence. The articles were classified as high, medium, and low evidence if they scored seven or more, between four and six, and under four, respectively.

Question	Interpretation
Was the primary objective of the study to estimate the incidence or prevalence of cSDH?	Yes: +1 point No: 1 point
What was the study design? Cross-sectional, retrospective, or prospective?	If retrospective: 1 point
How was cSDH defined?	All relevant ICD codes used: +1 point Not all relevant ICD codes used: 0.5 point Based on radiological findings: +1 point Not specified: 1 point
Sampling methodology	Sampling subjects from the general population: +1 point Nongeneral sampling methods (if self-reported by patients themselves or by surgeons): 0.5 point Nongeneral population-based sampling frame (medical records, healthcare based): 0.5 point
Is there a sampling bias? If so, was it addressed?	If existing and addressed: 0.5 point If existing but not addressed: 1 point
How was cSDH identified or confirmed in the population?	Reported with diagnosis method (CT scan): +1 point Obtained from medical records or administrative databases of the hospital or from registries: 0.5 point Not specified: 1 point
Were patients who died in hospital included? Were autopsies included?	Included: +1 point Not specified: 1 point
How was the incidence reported?	In cases/100′000/year: +1 point In % with the covered population provided: 0.5 point In % without the covered population provided: 1 point In cases/year with the covered population provided: 0.5 point In cases/year without the covered population provided: 1 point
Is the precision of the estimate reported? (SD or IC95 %)	Not reported: 0.5 point

Some of the included articles on chronic subdural hematoma also reported information on International Classification of Diseases (ICD) codes. All relevant ICD-9 and ICD-10 codes are summarized in Table 2. Where applicable, when the total number of cases over a given period was provided without a corresponding incidence rate, we estimated the incidence using the following formula: Incidence = (Number of new cases)/(Population × Timeframe).

**Table 2:** ICD-codes (International Classification of Diseases) ICD-codes deemed relevant to define chronic subdural hematoma**. Abbreviations:** ICD, International Classification of Diseases.

ICD Codes	Description
**ICD-9 codes**	
432.1	Subdural hemorrhage (nontraumatic)
852.2	Subdural hemorrhage following injury without mention of open intracranial wound
852.3	Subdural hemorrhage following injury with open intracranial wound
**ICD-10 codes**	
I62.0	Nontraumatic subdural hemorrhage
S06.5	Traumatic subdural hemorrhage

## 3 Results

The combined search yielded a total of 6253 abstracts, and 6187 were excluded based on abstract. Sixty-six articles were assessed for eligibility, and 39 were finally excluded after full-text analysis. We excluded articles if they did not specify cSDH, if the article was based on a sub-population, if the sample size was inferior to 100 patients and if the population was based on a single centre without providing the covered population. Eighteen articles ultimately met the inclusion criteria (Fig. 1). Among these, 7 discussed surgical incidence, and 11 provided incidence based on imaging or clinical findings, underscoring the variability in how cSDH is defined. All of these articles, reported the incidence on a regional, provincial/state, or national level (or sufficient information to compute it). However, none reported prevalence. Articles that did not meet our final inclusion criteria but provided important demographic trends were further reviewed.

To extract data, most studies used a retrospective analysis of medical records. Most did not specify how they defined cSDH, but five of them defined the condition with ICD codes. Table 3a, Table 3ba and 3b, Table 4a, Table 4bb report detailed information about the incidence studies and studies kept for demographics.

**Table 3a:** Incidence studies – Period, location, data source and case definition.

First author and level of evidence	Incidence period	Location	Data source	Case definition
Tommiska et al. (Tommiska et al., 2022) Medium	1997–2014	Finland	Finnish Care Register for Health Care	ICD-10: S06.5 or I62.0 and NOMESCO (Nordic Medico-Statistical Committee) Classification of Surgical Procedures Finland code of AAD10
Rauhala et al. (Rauhala et al., 2020a) Medium	1990–2015	Pirkanmaa Region, Finland	Patient administrative databases of the hospital The official Cause of Death Register, coordinated by Statistics Finland (Helsinki, Finland)	ICD-9 : 432.1, 852.2, and 852.3 ICD-10: S06.5 and I62.0 Exclusion criteria: acute or subacute SDH (<3 weeks after head trauma), hygroma (a collection of subdural CSF without signs of blood), and any form of intracranial surgery within 12 months preceding the CSDH diagnosis
Rauhala et al. (Rauhala et al., 2020b) Medium	1990–2015	Pirkanmaa Region, Finland	Patient administrative databases of the hospital Annual number of inhabitants in the Pirkanmaa Region: Statistics Finland (Helsinki, Finland)	ICD-10 codes: S06.5 and I62.0 ICD-9 codes: 432.1, 852.2 and 852.3
R. Fogelholm and O. Waltimo (Fogelholm and Waltimo, 1975) Low/Medium	1967–1973	Helsinki, Finland	During life: not specified Autopsy: files	Diagnosis during life: hematoma contained within well formed membranes Diagnosis on autopsy: hematomas which were brown, yellow, or grey in colour, attached to the dura, measured more than 5 mm in thickness
P. Mellergård and O. Wisten (Mellergard and Wisten, 1996) Low	1969; 1979; 1989; 1993	Lund, Sweden	Medical records	Not specified, based on angiography and CT-scans
Bartek et al. (Bartek et al., 2017) Low	2005–2010	Stockholm, Sweden Tromsø, Norway Trondheim, Norway	Electronic medical charts Data from Statistics Norway (www.ssb.no) Data from Statistics Sweden (www.scb.se)	Not specified
Asghar et al. (Asghar et al., 2002) Medium	1997–1999	North Wales, United Kingdom	Clinical notes and information form inpatient and outpatient notes Population aged 65 and above: Statistical directorate, National Assembly of Wales	ICD codes: codes not provided
Adhiyaman et al. (Adhiyaman et al., 2017) Medium	2014–2015	North Wales, United Kindgom	Radiology reports of patients above the age of 65 with CSDH from radiology reports on CT head scans	Not specified, based on radiological diagnosis
Stubbs et al. (Stubbs et al., 2021) Medium	2015–2018	East England (Cambridgeshire, Suffolk, Bedfordshire, and Norfolk), United Kingdom	Cases: not specified Population estimates: Office for National Statistics (2012) 2011 Census	Not specified
Grassner et al. (Grassner et al., 2021) Low	March–April 2020 2017–2019: for reference	Austria, Czech Rep., Switzerland	Not specified	cSDH requiring surgical evacuation due to the mass effect and/or neurological symptoms
Balser et al. (Balser et al., 2015) Medium/High	2000–2012	United States of America	New York Harbor Healthcare System VISTA (Veterans Health Information Systems and Technology Architecture) network	Not specified, based on radiological diagnosis
Toi et al. (Toi et al., 2018) Low	2010–2013	Japan	DPC (Diagnostic Procedure Combination) administrative national database which reports information about acute inpatient care and payment	ICD-10 code of S06.5
Kudo et al. (Kudo et al., 1992) Low	1986–1988	Awaji Island, Hyogo prefecture, Japan	Not specified	Not specified
Karibe et al. (Karibe et al., 2011) Low/Medium	2005–2007	Miyagi prefecture, Japan	Miyagi Traumatic Head Injury Registry Project including the 17 hospitals with neurosurgery ward in the Miyagi prefecture	CT scan: isodense collections as subacute hematomas and hypodense as chronic hematomas
Nayil et al. (Nayil et al., 2012) Low	January 2006– December 2009	Sher-i-Kashmir Institute of Medical Sciences, Kashmir, India (only hospital treating neurosurgical patients in the region)	Medical records	Not specified
Rust et al. (Rust et al., 2006) Low	1996–2001	Royal Hobart Hospital, Tasmania, Australia	cSDH: medical records Estimated resident population in Tasmania: Australian Bureau of Statistics about the population in Tasmania Warfarin usage: Health Insurance Commission Information Services Branch	Not specified, based on radiological diagnosis
Magalhães et al. (Magalhães et al., 2019) Medium	2008–2016	Brazil	Database of the Brazilian Unified Health System, issuing hospital admission authorizations (HAAs) at the Informatics Department of the Unified Health System (DATASUS)	Code for HAAs at the DATASUS:04.03.01.03.14 (surgical treatment of chronic subdural hematoma)
Cousseau et al. (Cousseau et al., 2001) Medium	1992–1996	Hospital Privado de Comunidad de Mar del Plata, Argentina	Medical charts of patients in neurosurgery and radiology wards	Not specified, based on radiological diagnosis No differentiation of cSDH and saSDH (difficult to distinguish on radiological findings)

**Abbreviations:** ICD, International Classification of Disease; CSF, cerebro-spinal fluid; SDH, subdural hematoma; cSDH, chronic subdural hematoma, saSDH, sub-acute subdural hematoma.

**Table 3b:** – Studies kept for analysis of Demographics.

First author and level of evidence	Period	Location	Data Source	Case definition
Illy e. et al. (Illy et al., 2020) Low	2005–2012	Münster, Deutschland	Not specified	Not specified
Baechli et al. (Baechli et al., 2004) Low	1996–2002	Basel, Switzerland	Medical records	Not specified, based on radiological diagnosis
Iihara et al. (Iihara et al., 2021) Low	2018–2019	Japan	Japan Neurosurgical Database which is a prospective observational study registry that aims to collect all information related to inpatient treated in neurosurgical departments of hospitals included in the Japan Neurosurgery Society	Not specified
Sambasivan M. (Sambasivan, 1997) Low	1966–1996	Cosmopolitan Hospital Trivandrum, Kerala State, India	Not specified	Not specified, based on radiological diagnosis
Sousa et al. (Sousa et al., 2013) Low	May 2014–April 2015	Hospital de Base do Distrito Federal, Brasília, Brazil	Medical records	CT scan: crescent-shaped hypodense or isodense hemispheric collection of blood layered over the cerebral convexity, independent of knowledge of an occurrence of a traumatic injury
Idowu et al. (Idowu et al., 2021) Low	January 2004 and July 2014	Lagos State University teaching Hospital, Ikeja, Lagos, Nigeria	Medical records	Not specified

**Table 4a:** – Incidence studies – Annual incidence, M:F ratio, age of highest incidence, mean age, causes.

First author	Annual incidence	M/F ratio	Age of highest incidence	Mean age	Cause
Tommiska et al. (Tommiska et al., 2022)	1997–2002 : 12.1/100′000/year 2012–2014 : 16.5/100′000/year *Surgical incidence*	2:1	90+	F: 78 M: 73 1997 : 73 (61–80) 2014 : 76 (67–83)	Not specified
Rauhala et al. (Rauhala et al., 2020a)	1990–1995 : 8.2/100′000 2010–2015 : 17.6/100′000	1.9:1 (65 % M)	90+	73 years (1990–1995) to 79 years (2011–2015)	Head trauma: 59 % Bicycle accidents: 23.3 % Fall from 1m: 23.3 % OH-related (1990–1995): 16 % OH-related (2011–2015): 7 % Chronic OH abuse Anticoagulants
Rauhala et al. (Rauhala et al., 2020b)	Incidence of operatively treated cSDH: −1990–1995 : 7.2/100′000/year −2011–2015 : 13.4/100′000/year Non operative ≥80 years: −2011–2015 : 36.9/100′000/year	1.9:1 (65 % M)	Not specified	Operative and non operative: 76	In operated cases: - Trauma: 59.1 % (679/1148)
R. Fogelholm and O. Waltimo (Fogelholm and Waltimo, 1975)	1.72/100,000/year *Surgical incidence*	2.3:1 (30 % F)	70–79	Not specified	Autopsy: trauma 46 %
P. Mellerghård and O. Wisten (Mellergard and Wisten, 1996)	1969 : 2.08/100′000/year 1979 : 2.88/100′000/year 1989 : 4.41/100′000/year 1993 : 5.32/100′000/year *Surgical incidence*	2.6:1 (27.6 % F)	Not specified	70.5	Trauma: 62 % Alcoholism: 14.7 % Anticoagulants: 10.8 %
Bartek et al. (Bartek et al., 2017)	18-49 : 0.6/100′000 50-66 : 7.1/100′000 67-79 : 27.3/100′000 80-89 : 59.5/100′000 >90 : 52.0/100′000 *Surgical incidence: burr-hole evacuation only*	Not specified	80–89	>90 years old group: 92.2 ± 1.9 <90 years old group: 72.8 ± 11.9	Not specified
Asghar et al. (Asghar et al., 2002)	>65 ans: 8.2/100′000/an	1.9:1 (35 % F)	Not specified	79 (65–84)	Falls: 57 % No risk factor: 10 %
V. Adhiyaman et al. (Adhiyaman et al., 2017)	>65 years old: 48/100′000/year	1:1.6	95+	81 years old	50 %: falls
Stubbs et al. (Stubbs et al., 2021)	Surgical incidence: 3.50/100′000/year (3.19–3.85) Directly standardised incidence: 1.58/100′000/year (1.26–1.90)	Not specified	80–89	Not specified	Not specified
Grassner et al. (Grassner et al., 2021)	Austria: (P-value = 0.01) 2017 : 72 cases/month 2018 : 63cases/month 2019 : 62 cases/month 2020 : 38 cases/month Czech Republic, : (P-value = 0.002) 2017 : 87 cases/month 2018 : 110 cases/month 2019 : 84 cases/month 2020 : 53 cases/month Switzerland: 2017 : 38 cases/month 2018 : 37 cases/month 2019 : 36 cases/month 2020 : 41 cases/month *Non-elective neurosurgical cases during COVID-19*	All neurosurgical cases: Female = 44 % (AU and CH), 41 % (Czech rep.)	Not specified	All neurosurgical cases: Austria and the Czech Rep. : 61 Switzerland: 65	Not specified
Balser et al. (Balser et al., 2015)	Veteran population: 79.4/100,000 Age-standardized: 39.1/100,000 ± 4.74	4.6:1	80–84 years	Not specified	Not specified
Toi et al. (Toi et al., 2018)	16.6/100′000/person-year	Not specified	Not specified	Not specified	Not specified
Kudo et al. (Kudo et al., 1992)	≥ 65 years: 8.6–153.5/100′000/year (mean: 58.1) <65 years: mean 3.4/100′000/year Total incidence: 13.1/100′000 (4.0–36.1/100′000 p.y)	Not specified	Not specified	Not specified	Not specified
Karibe et al. (Karibe et al., 2011)	20.6/100′000 p.y	2.4:1	+80 years	71.2 ± 12.8 years	Fall (ground level):46.7 % Fall (height): 7.4 % Traffic accident: 13.8 % Violence: 1.8 % Sports and others: 4.8 % Unknown: 25.5 %
Nayil et al. (Nayil et al., 2012)	3,5/100′000 person-year	3.3:1	Not specified	60.4 ± 12.4	Not specified
Rust et al. (Rust et al., 2006)	Warfarin group: 80/100′000 p.y No anticoagulation group: 2/100′000 p.y	2.4:1 (29.6 %F)	Not specified	Not specified	Fall/light bump to the head: 59.3 % Motor vehicle accident: 4.9 % VP shunt: 7.4 % No history of trauma: 27.1 %
Magalhães et al. (Magalhães et al., 2019)	2015 : 2.39/100,000 (hospital admissions) Supplementary material 2 for further data	Not specified	Not specified	Not specified	Not specified
Cousseau et al. (Cousseau et al., 2001)	Annual overall rate:14.1 CSSH/100,000 p.y Specific rate for women: 11.6 CSSH/100,000 p.y Specific rate for men: 18.1 CSSH/100,000 p.y Adjusted rate for the Argentinian population based on the national census of 1991 : 3.1/100′000p.y	Not specified	Not specified	66 years old (47–76 years)	51 %: TBI

**Abbreviations**: M, male; F, female; p.y, person/year; AU, Austria, CH, Switzerland; TBI, traumatic brain injury; VP, ventriculo-peritoneal.

Ten studies reported cSDH incidence in a European country or region. In these 10 articles, 7 reported surgical incidence. Two additional studies did not meet our final inclusion criteria, but we still analysed their demographics data.

No study reported national data on clinical incidence, and only one study provided national data on surgical incidence of cSDH (Finland). The age-standardized surgical incidence reached 12.2/100,000/year in 1997–2002, and increased up to 16.5/100,000/year in 2012–2014(12).

On a regional level, the highest age-standardized crude-incidence was found in the Pirkanmaa region of Finland, culminating at 17.6/100,000/year in 2010–2015(13). Regarding surgical incidence, it ranged from 1.58/100,000/year in 2015–2018 (East of England, United Kingdom) (Stubbs et al., 2021) to 5.32/100,000/year in 1993 (Lund, Sweden) (Mellergard and Wisten, 1996).

Only one study reported the incidence of cSDH in the North American continent from a US veteran population using a national database between 2000 and 2012 (Balser et al., 2015). The reported age-standardized incidence reached 39.1/100,000 ± 4.74 (Balser et al., 2015). In this report, a projected incidence rate of cSDH for 2030 in USA's veteran population was expected to be 121.4/100,000 (Balser et al., 2015).

Four articles reported the incidence of cSDH in Asian countries. In Japan, from April 2010 to March 2013, 63,358 patients were newly diagnosed with cSDH (Toi et al., 2018). The population in 2010 was 128.1 million (Statistics Bureau Home Page), resulting in an overall estimated incidence of 16.6/100,000 person-year (Tenny and Boktor, 2023). Moreover, from 2018 to 2019, there was an increase in the number of patients, and represented the most frequent diagnosis in the field (Iihara et al., 2021). Regionally in Japan, the lowest incidence was calculated in Awaji region in 1986–1988 and reached 13.1/100,000/year. According to their findings, the national incidence during this time should be 16.3/100,000/year (Kudo et al., 1992). The highest was reported in Miyagi Prefecture and reached 20.6/100,000/year in 2005–2007 (Karibe et al., 2011). In the Jammu and Kashmir region in India, 1316 new cases of cSDH were diagnosed between January 2006 and December 2009 (Nayil et al., 2012). In this region, the population in 2011 was 12,541,302 (Jammu and Kashmir Population, 2022), providing a grossly estimated incidence of 3.5/100,000 person-year. The population in 2006 and 2009 was not available.

One article reported the incidence between the population with or without anticoagulation therapy in Oceania, Tasmania, Australia (Rust et al., 2006). Over a five-year period, from 1996 to 2001, the incidence of cSDH in the population that does not use anticoagulation reached 2/100,000/year, whereas in the population using warfarin, it reached 80/100,000/year, with a combined incidence of 82/100,000/year.

Two articles reported the incidence of cSDH in South America, one from Brazil and one from Argentina. In Brazil, the incidence was 2.39/100,000/year in 2015 based on cross-multiplication (Magalhães et al., 2019). We report the incidence of hospitalization for each year in Supplementary material 2. In the city of Mar del Plata (Argentina), the overall incidence of cSDH was 14.1/100,000/year in the years 1992–1996 (CI 95 % 10.6–17.6) (Cousseau et al., 2001). However, their facility provides care primarily to people aged 65 years and above, which should be taken into consideration before interpreting this estimate. The author adjusted their findings to the National Population Census of Argentina in 1991 and estimated a national incidence of 3.1/100,000/year (Cousseau et al., 2001).

No articles reported the incidence of cSDH in Africa, but one article reported demographics from 2001 to 2009, in Lagos (Nigeria) (Idowu et al., 2021).

Table 4a, Table 4ba and 4b provide detailed information on overall incidence and surgical incidence, while Supplementary Material 1 presents age- and sex-stratified incidence data.

**Table 4b:** – Studies kept for demographics.

First Author	M:F	Age of highest incidence	Mean age	Causes
Illy e. et al. (Illy et al., 2020)	2.3:1 (185:83) (65 % M)	Not specified	74	Not specified
Baechli et al. (Baechli et al., 2004)	Younger group (<65) = 2.3:1 Older group (>65) = 1.6:1	≥65years (69 %)	68.3 years (SD ± 17.0)	Falls: 77 %
Iihara et al. (Iihara et al., 2021)	Not specified	Not specified	Not specified	Trauma
Sambasivan M. (Sambasivan, 1997)	5:1	41–50 years	Not specified	Trauma: 9 %
Sousa et al. (Sousa et al., 2013)	4.8:1	Not specified	Overall: 64.3 ± 15.9 Males: 63.0 ± 14.9 Females: 70.0 ± 18.8	Not specified
Idowu et al. (Idowu et al., 2021)	2:1 (M:F)	7th decade of life	Males: 61 Females: 62	Not specified

**Abbreviations:** M, Male; F, Female; SD, standard deviation.

Most studies reported a higher clinical and surgical incidence among elderly patients, with nearly all showing an increase over time in older age groups. In addition, almost all regions demonstrated a higher male-to-female ratio, and the predominant causes of cSDH were trauma or falls.

## 4 Discussion

cSDH is one of the most common neurosurgical conditions encountered in daily practice and predominantly affects the elderly. Understanding its incidence and prevalence is essential for designing preventive strategies targeted to at-risk populations and for evaluating their impact on healthcare costs. Monitoring epidemiological trends is similarly crucial for assessing the effectiveness of these interventions over time.

Despite an extensive systematic review, no prevalence data regarding cSDHs were available. However, eighteen studies reported clinical or surgical incidence: ten in Europe, one in North America, four in Asia, one in Oceania, two in South America, and none in Africa. These studies showed that the incidence rates of cSDH varies substantially, for example from 8.2/100,000/year in Pirkanmaa region (Finland) to 39.1/100,000 in the USA. Despite this, across studies the incidence of cSDH was consistently higher in the elderly, more common in males, and showed a rising trend over time. The increasing incidence of cSDH in recent years is likely due to prolonged life expectancy as well as improved access to diagnostic tools such as imaging technology. This may likewise contribute to the lower incidence observed in less affluent countries, such as Brazil. However, the paucity of available data prevents firm conclusions. The most common aetiology of cSDH was found to be traumatic brain injury or other accidents that frequently result in cranial trauma such falls, and motor vehicle accidents.

Given that falls occur more frequently in older adults, it is expected that the elderly are at greater risk of cSDH, even when the fall results in only modest head injury.

Another important contributing factor is antithrombotic medication use, which can result in spontaneous bleeding or facilitate bleeding after relatively minor injury. The rising use of antithrombotic medications, particularly among the elderly, is very likely contributing to the increased incidence observed in older patients.

Finally, alcohol consumption is an important and preventable risk factor for cSDH, acting through multiple mechanisms. Alcohol abuse increases the likelihood of accidents (Iyer et al., 2022) and can also lead to coagulopathies and craniocephalic disproportion (brain atrophy) with chronic use (Yang et al., 2012; Paul et al., 2008) (Mukamal et al., 2001).

Despite recognition of these and other risk factors, approximately 25 % of patients still present with cSDH of unknown aetiology. Although understanding of the pathophysiology continues to improve, unidentified risk or predisposing factors may also contribute to the development of the disease.” The recent hypothesis that the middle meningeal artery plays a key role in both the pathophysiology and recurrence of cSDH may offer additional insight into the natural history of the condition. Indeed, not all patients sustaining a traumatic brain injury develop cSDH (Izumihara et al., 2013), suggesting that other factors are at play. It has been proposed that injury to the dural border cells triggers an inflammatory response that induces angiogenesis, leading to the formation of fragile, permeable vessels and subsequent fluid accumulation. This mechanism may also apply to patients without a history of traumatic brain injury, suggesting that additional, yet-unidentified factors capable of damaging dural border cells need to be investigated (Nouri et al., 2021; Edlmann et al., 2017b).

The most important epidemiological trend highlighted by this review is the rising incidence of cSDH across multiple regions, particularly among the elderly. The reasons for this increase are multifactorial. The implications are substantial, both medically and economically, as healthcare costs related to cSDH are expected to grow and should be anticipated. This underscores the need for implementing treatment strategies that are both effective and cost-efficient.

Despite the emergence of a promising new strategy—middle meningeal artery embolization—it remains unclear whether this approach is effective as a standalone treatment or only in combination with surgery to reduce recurrence. Its cost-effectiveness also has yet to be established (Feghali et al., 2020). To this end, our group has launched a randomized controlled trial, with results to be announced over the next two years (Al Awadhi et al., 2025).

As noted, traumatic brain injury from falls is one of the most common causes of cSDH. The prevalence of falls among the elderly is nearly 27 %, and the associated economic burden on healthcare is substantial (Salari et al., 2022; Heinrich et al., 2010). Strengthening fall-prevention strategies is therefore an important measure to reduce the incidence of cSDH in this age group. In addition, the rising use of antithrombotic medications must be taken into account. Their prescription and adjustment should be carefully monitored, and preference should be given to blood thinners that are easier to manage. Finally, public health initiatives aimed at reducing harmful alcohol consumption may meaningfully lower the long-term burden of cSDH, given the well-established link between chronic alcohol abuse and increased risk of both traumatic and non-traumatic intracranial bleeding.

It is important to note that systematic reviews of epidemiological studies have important limitations. First, there remains a possibility that some relevant evidence was not captured, which could affect the completeness and validity of this review (Dickersin, 2002; Uttley et al., 2023). To mitigate this, we included studies published in any language and designed our search strategy to be as inclusive as possible. A second limitation relates to the methodological rigor of the included studies. Many did not formally assess risk of bias, which diminishes the overall reliability and strength of the evidence synthesized in this review (Dickersin, 2002; Uttley et al., 2023). The discrepancies in incidence rates reported across regions may stem from differences in how cases were identified and defined, the characteristics of the populations studied, and the unequal availability of diagnostic tools between high- and low-income countries.

Regarding case definition, many studies did not specify how cSDH was identified. Even when ICD codes were reported, the codes used varied across studies. This poses an additional challenge, as ICD classifications typically distinguish only between traumatic and non-traumatic subdural hematomas, making it difficult to isolate chronic cases specifically (Langlois et al., 2023). Despite our efforts to include only studies focused on cSDH, it is likely that some articles also encompassed acute or subacute subdural hematomas, introducing a potential source of bias in the estimation of incidence.

Falls and other trauma often prompt neuroimaging when a traumatic brain injury is suspected, which increases the likelihood of detecting cSDH in these situations. In contrast, non-traumatic cSDH is likely underdiagnosed, as it may present subtly and does not routinely trigger imaging. As a result, epidemiological studies likely underestimate the true incidence of the disease.

Here, we included articles providing surgical incidence. Many European studies focused primarily on surgical rather than overall incidence. These findings must be interpreted with caution, as a substantial proportion of individuals with cSDH remain asymptomatic and never seek surgical care. This may lead to an underestimation of surgical incidence and an overestimation of general incidence. These factors, combined with the lack of standardized data collection across studies, limit the comparability of reported incidence figures. Moreover, despite attempts to extract prevalence data, none of the included studies provided clear prevalence estimates. A further limitation of this review is the lack of consistent, country-specific demographic data such as life expectancy and age distribution. This prevents a more accurate evaluation of the relationship between population aging and cSDH incidence.

Despite these limitations and the scarcity of epidemiological studies, this review represents the first systematic effort to compile global epidemiological data on cSDH. A major strength of our work lies in the breadth of our search strategy, which included studies in all languages and without geographic restrictions, allowing us to capture data from across the world.

## 5 Conclusion

The present review demonstrates clear evidence that the incidence of cSDH has been rising over time across multiple regions, driven by factors such as population aging, traumatic brain injuries, and the widespread use of antithrombotic medications. Despite these advances, major gaps remain in our understanding of the global burden of cSDH, particularly due to the lack of prevalence data and inconsistencies in data collection methods.

As the incidence is expected to continue increasing alongside aging demographics, the associated economic burden will likely grow as well. Future epidemiological research is therefore essential to more accurately quantify the disease burden, refine preventive strategies, and develop cost-effective treatment approaches that address both the medical and financial challenges posed by cSDH.

## Contributors

AD and AN conceptualized and designed the study. AD, ER, RS and AF acquired the data. AD, ER and AN analysed and interpreted the data. AD, AA, CM, SG and AN drafted the original manuscript. AA, CM, SG, and AN critically reviewed. CM, SG, AA and AD extensively edited the article for submission. AN, SG and KS supervised the study. All authors had full access to all the data and had final responsibility for the decision to submit for publication.

## Data sharing

All the data collected for the study, including the data dictionary and the statistical analyses, will be made available via email enquiry upon publication of this study. The study protocol and the statistical analysis plan will also be made available at the same time. The data will be supplied to researchers after approval with a signed data access agreement.

## Funding statement

This research received no specific grant from any funding agency in the public, commercial or not-for-profit sectors.

## Declaration of competing interest

The authors declare that they have no known competing financial interests or personal relationships that could have appeared to influence the work reported in this paper.

## References

[bib1] Adhiyaman V., Chattopadhyay I., Irshad F., Curran D., Abraham S. (2017). Increasing incidence of chronic subdural haematoma in the elderly. QJM..

[bib2] Al Awadhi A., Mollica C., Da Broi M., Molliqaj G., Hofmeister J., Rosi A., Bernava G., Machi P., Morel S., Cardia A., Meling T.R., Schaller K., Nouri A. (2025). Middle meningeal artery (MMA) embolisation for chronic subdural haematomas: rationale and design for the STOp recurrence of MMA bleeding (STORMM) randomised control trial-a study protocol. BMJ Open.

[bib3] Asghar M., Adhiyaman V., Greenway M.W., Bhowmick B.K., Bates A. (2002). Chronic subdural haematoma in the elderly--a north Wales experience. J. R. Soc. Med..

[bib4] Baechli H., Nordmann A., Bucher H.C., Gratzl O. (2004). Demographics and prevalent risk factors of chronic subdural haematoma: results of a large single-center cohort study. Neurosurg Rev [Internet].

[bib5] Balser D., Farooq S., Mehmood T., Reyes M., Samadani U. (2015). Actual and projected incidence rates for chronic subdural hematomas in United States veterans administration and civilian populations. J. Neurosurg..

[bib6] Bartek J., Sjåvik K., Ståhl F., Kristiansson H., Solheim O., Gulati S. (2017). Surgery for chronic subdural hematoma in Nonagenarians: a Scandinavian population-based multicenter study. Acta Neurol. Scand..

[bib7] Chen J.C., Levy M.L. (2000). Causes, epidemiology, and risk factors of chronic subdural hematoma. Neurosurg Clin N. Am.

[bib8] Cousseau D.H., Echevarría G.M., Gaspari M., Gonorazky S.E. (2001). Hematoma subdural crónico y subagudo. Estudio epidemiológico en una población cautiva. Rev. Neurol..

[bib9] Dickersin K. (2002). Systematic reviews in epidemiology: why are we so far behind?. Int. J. Epidemiol..

[bib10] Edlmann E., Giorgi-Coll S., Whitfield P.C., Carpenter K.L.H., Hutchinson P.J. (2017). Pathophysiology of chronic subdural haematoma: inflammation, angiogenesis and implications for pharmacotherapy. J. Neuroinflammation.

[bib11] Edlmann E., Giorgi-Coll S., Whitfield P.C., Carpenter K.L.H., Hutchinson P.J. (2017). Pathophysiology of chronic subdural haematoma: inflammation, angiogenesis and implications for pharmacotherapy. J. Neuroinflammation.

[bib12] Feghali J., Yang W., Huang J. (2020). Updates in chronic subdural hematoma: epidemiology, etiology, pathogenesis, treatment, and outcome. World Neurosurg. Sept.

[bib13] Fogelholm R., Waltimo O. (1975). Epidemiology of chronic subdural haematoma. Acta Neurochir..

[bib14] Grassner L., Petr O., Warner F.M., Dedeciusova M., Mathis A.M., Pinggera D. (2021). Trends and outcomes for non-elective neurosurgical procedures in central Europe during the COVID-19 pandemic. Sci. Rep..

[bib15] Heinrich S., Rapp K., Rissmann U., Becker C., König H.H. (2010). Cost of falls in old age: a systematic review. Osteoporos. Int..

[bib16] Hendrix P., Goren O., Dalal S., Kanmounye U.S., Weiner G.M., Schirmer C.M. (2022). In-hospital mortality rates, complication rates, length of stay, and total costs of >14,000 chronic subdural hematomas treated in the U.S. between 2016 and 2020: query of the premier health-care database. Surg. Neurol. Int..

[bib17] Idowu O.E., Vitowanu J.M., Oyeleke S.O. (2021). Demographic profile and outcome in surgically managed patients with chronic subdural haematoma: a 9-Year retrospective cohort study.

[bib18] Iihara K., Saito N., Suzuki M., Date I., Fujii Y., Houkin K. (2021). The Japan neurosurgical database: Statistics update 2018 and 2019. Neurol. Med.-Chir..

[bib19] Illy E., Gerss J., Fischer B.R., Stummer W., Brokinkel B., Holling M. (2020). Influence of meteorological conditions on the incidence of chronic subdural haematoma, subarachnoid and intracerebral haemorrhages - the « bleeding weather hypothesis. Turk Neurosurg.

[bib20] Iyer A., Killian M., Stead T.S., Mangal R., Ganti L. (2022). Acute-on-Chronic subdural hematoma secondary to falls due to alcoholism. Cureus. Sept.

[bib21] Izumihara A., Yamashita K., Murakami T. (2013). Acute subdural haematoma requiring surgery in the subacute or chronic stage. Neurol. Med.-Chir..

[bib22] Jammu and Kashmir Population (2022). Sex ratio & literacy rate 2023. https://www.census2011.co.in/census/state/jammu+and+kashmir.html.

[bib23] Karibe H., Kameyama M., Kawase M., Hirano T., Kawaguchi T., Tominaga T. (2011). [epidemiology of chronic subdural hematomas]. Noshinkeigeka.

[bib24] Kudo H., Kuwamura K., Izawa I., Sawa H., Tamaki N. (1992). Chronic subdural hematoma in elderly people: present status on awaji Island and epidemiological prospect. Neurol. Med.-Chir..

[bib25] Langlois A.M., Touchette C.J., Mathieu D., Iorio-Morin C. (2023). Classification of subdural hematomas: proposal for a new system improving the ICD coding tools. Front. Neurol..

[bib26] Magalhães MJ da S de, Araújo J.P., Paulino A.L.A.S.A., Batista B.H.M., Freitas DG de, Santos JD. da C. (2019). Epidemiology and estimated cost of surgery for chronic subdural hematoma conducted by the unified health system in Brazil (2008–2016). Arq Bras Neurocir Braz Neurosurg.

[bib27] Mellergard P., Wisten O. (1996). Operations and re-operations for chronic subdural haematomas during a 25-year period in a well defined population. Acta Neurochir..

[bib28] Mukamal K.J., Jadhav P.P., D'Agostino R.B., Massaro J.M., Mittleman M.A., Lipinska I. (2001). Alcohol consumption and hemostatic factors: analysis of the framingham offspring cohort. Circulation. 18 Sept.

[bib29] Nayil K., Ramzan A., Sajad A., Zahoor S., Wani A., Nizami F. (2012). Subdural hematomas: an analysis of 1181 Kashmiri patients. World Neurosurg..

[bib30] Nouri A., Gondar R., Schaller K., Meling T. (2021). Chronic subdural hematoma (cSDH): a review of the current state of the art. Brain Spine.

[bib31] Paul C.A., Au R., Fredman L., Massaro J.M., Seshadri S., Decarli C. (2008). Association of alcohol consumption with brain volume in the framingham study. Arch. Neurol..

[bib32] Rauhala M., Luoto T.M., Huhtala H., Iverson G.L., Niskakangas T., Öhman J. (2020). The incidence of chronic subdural hematomas from 1990 to 2015 in a defined Finnish population. J. Neurosurg..

[bib33] Rauhala M., Helén P., Huhtala H., Heikkilä P., Iverson G.L., Niskakangas T. (2020). Chronic subdural hematoma—Incidence, complications, and financial impact. Acta Neurochir..

[bib34] Regan J.M., Worley E., Shelburne C., Pullarkat R., Watson J.C. (2015). Burr hole washout versus craniotomy for chronic subdural hematoma: patient outcome and cost analysis. PLoS One.

[bib35] Rust T., Kiemer N., Erasmus A. (2006). Chronic subdural haematomas and anticoagulation or anti-thrombotic therapy. J. Clin. Neurosci..

[bib36] Sahyouni R., Goshtasbi K., Mahmoodi A., Tran D.K., Chen J.W. (2017). Chronic subdural hematoma: a historical and clinical perspective. World Neurosurg..

[bib37] Salari N., Darvishi N., Ahmadipanah M., Shohaimi S., Mohammadi M. (2022). Global prevalence of falls in the older adults: a comprehensive systematic review and meta-analysis. J. Orthop. Surg. Res..

[bib38] Sambasivan M. (1997). An overview of chronic subdural hematoma: experience with 2300 cases. Surg. Neurol..

[bib39] Sim Y.W., Min K.S., Lee M.S., Kim Y.G., Kim D.H. (2012). Recent changes in risk factors of chronic subdural Hematoma. J. Korean Neurosurg. Soc..

[bib40] Singh A., Tetreault L., Kalsi-Ryan S., Nouri A., Fehlings M.G. (2014). Global prevalence and incidence of traumatic spinal cord injury. Clin. Epidemiol..

[bib41] Sousa E.B., Brandão L.F., Tavares C.B., Borges I.B., Neto N.G.F., Kessler I.M. (2013). Epidemiological characteristics of 778 patients who underwent surgical drainage of chronic subdural hematomas in brasília, Brazil. BMC Surg..

[bib42] Statistics bureau home Page/POPULATION AND HOUSEHOLDS OF JAPAN 2010. https://www.stat.go.jp/english/data/kokusei/2010/final_en/final_en.html#Summary.

[bib43] Stubbs D.J., Vivian M.E., Davies B.M., Ercole A., Burnstein R., Joannides A.J. (2021). Incidence of chronic subdural haematoma: a single-centre exploration of the effects of an ageing population with a review of the literature. Acta Neurochir..

[bib44] Tenny S., Boktor S.W. (2023). StatPearls [Internet].

[bib45] Toi H., Kinoshita K., Hirai S., Takai H., Hara K., Matsushita N. (2018). Present epidemiology of chronic subdural hematoma in Japan: analysis of 63,358 cases recorded in a national administrative database. J. Neurosurg..

[bib46] Tommiska P., Luostarinen T., Kaprio J., Korja M., Lönnrot K., Kivisaari R. (2022). Incidence of surgery for chronic subdural hematoma in Finland during 1997–2014: a nationwide study. J. Neurosurg..

[bib47] Uttley L., Quintana D.S., Montgomery P., Carroll C., Page M.J., Falzon L. (2023). The problems with systematic reviews: a living systematic review. J. Clin. Epidemiol..

[bib48] Welling L.C., Welling M.S., Teixeira M.J., Figueiredo E.G. (2018). Chronic subdural hematoma: so common and So neglected. World Neurosurg..

[bib49] Yadav Y., Parihar V., Namdev H., Bajaj J. (2016). Chronic subdural hematoma. Asian J. Neurosurg..

[bib50] Yang A.I., Balser D.S., Mikheev A., Offen S., Huang J.H., Babb J. (2012). Cerebral atrophy is associated with development of chronic subdural haematoma. Brain Inj..

